# P-1808. Strategies for Antimicrobial Stewardship Program Resiliency During the COVID-19 Pandemic

**DOI:** 10.1093/ofid/ofae631.1971

**Published:** 2025-01-29

**Authors:** Rebecca Schwei, Meggie Griffin, Helena Ikenberry, Ashleen Kaur, Sarah Scalzo, Aurora E Pop-Vicas, Lucas Schulz, Nicole Werner, Michael Pulia

**Affiliations:** University of Wisconsin-Madison, Madison, Wisconsin; University of Wisconsin - Madison, Madison, Wisconsin; University of Wisconsin Madison, MADISON, Wisconsin; University of Wisconsin Madison, MADISON, Wisconsin; University of Wisconsin Madison, MADISON, Wisconsin; University of Wisconsin School of Medcine and Public Health, Madison, WI; University of Wisconsin Hospital and Clinics, Madison, Wisconsin; Indiana University School of Public Health-Bloomington, Bloomington, Indiana; University of Wisconsin-Madison, Madison, Wisconsin

## Abstract

**Background:**

The COVID-19 pandemic was an unprecedented stress test for hospital-based antimicrobial stewardship (AMS) programs. The purpose of this study was to characterize strategies for AMS program resiliency during the COVID-19 pandemic.

**Figure:**
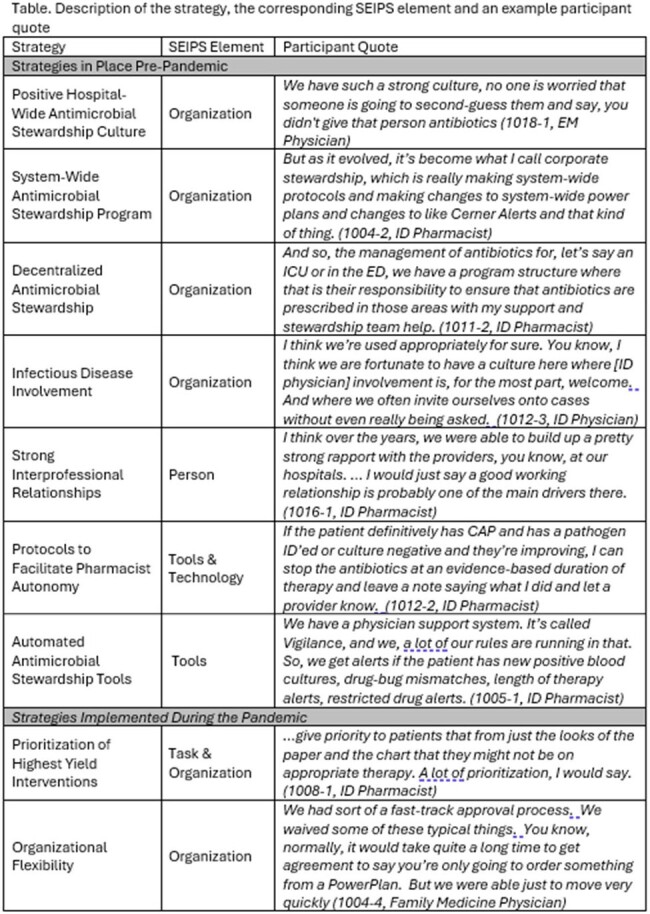

**Methods:**

Using a national dataset, we identified hospitals according to three criteria: 1.) significant COVID-19 burden; 2.) appropriate antibiotic prescribing patterns before the pandemic; and 3.) maintenance of appropriate antibiotic prescribing patterns during the pandemic. We conducted semi-structured interviews with pharmacists, physicians and quality leaders involved in AMS during the pandemic from identified hospitals. Interview guides were developed using the Systems Engineering Initiative for Patient Safety (SEIPS) framework. Transcribed interviews were analyzed iteratively using content analysis directed by the SEIPS model.

**Results:**

We conducted 30 interviews from 19 unique high-performing health care systems across the country. We interviewed 17 pharmacists, 12 physicians and 1 infection prevention nurse. Resilient AMS strategies in place pre-pandemic included positive hospital wide AMS culture (organization); system-wide AMS programs (organization); decentralized antimicrobial stewardship (organization); strong interprofessional relationships (organization & person); infectious disease involvement (person); protocols to facilitate pharmacist autonomy (organization & tools and technology) and automated tools to facilitate AMS program activities (tools and technology). Additionally, the following strategies were implemented during the pandemic to withstand increased workload and the rapidly evolving pandemic: prioritization of highest yield stewardship interventions (organization & task); and organizational flexibility (organization).

**Conclusion:**

Using a systems engineering informed qualitative approach, we characterized work system strategies to promote resiliency among AMS programs during the COVID-19 pandemic which should inform future efforts to promote AMS program resiliency during periods of health system stress.

**Disclosures:**

**All Authors**: No reported disclosures

